# Early and Prolonged Antiretroviral Therapy Is Associated with an HIV-1-Specific T-Cell Profile Comparable to That of Long-Term Non-Progressors

**DOI:** 10.1371/journal.pone.0018164

**Published:** 2011-04-05

**Authors:** Cristina Cellerai, Alexandre Harari, Hans Stauss, Sabine Yerly, Anna-Maria Geretti, Anne Carroll, Thynn Yee, Jonathan Ainsworth, Ian Williams, John Sweeney, Andrew Freedman, Margaret Johnson, Giuseppe Pantaleo, Sabine Kinloch-de Loes

**Affiliations:** 1 Division of Immunology and Allergy, Centre Hospitalier Universitaire Vaudois and University of Lausanne, Lausanne, Switzerland; 2 Swiss Vaccine Research Institute, University of Lausanne, Lausanne, Switzerland; 3 Department of Immunology, University College London-Royal Free Campus, London, United Kingdom; 4 Laboratory of Virology, University Hospital Geneva and University of Geneva Medical School, Geneva, Switzerland; 5 Virology Department, University College London-Royal Free Campus, London, United Kingdom; 6 The Royal Free Centre for HIV Medicine-Ian Charleson Day Centre, Royal Free Hospital, London, United Kingdom; 7 Haemostasis and Haemophilia Department, Royal Free Hospital, London, United Kingdom; 8 Department of Infectious Diseases, North Middlesex Hospital, London, United Kingdom; 9 Centre for Sexual Health and HIV Research, University College London, London, United Kingdom; 10 Department of GU Medicine, Victoria Hospital, Blackpool, United Kingdom; 11 Department of Medicine, Cardiff University School of Medicine, Cardiff, United Kingdom; Massachusetts General Hospital, United States of America

## Abstract

**Background:**

Intervention with antiretroviral treatment (ART) and control of viral replication at the time of HIV-1 seroconversion may curtail cumulative immunological damage. We have therefore hypothesized that ART maintenance over a very prolonged period in HIV-1 seroconverters could induce an immuno-virological status similar to that of HIV-1 long-term non-progressors (LTNPs).

**Methodology/Principal Findings:**

We have investigated a cohort of 20 HIV-1 seroconverters on long-term ART (LTTS) and compared it to one of 15 LTNPs. Residual viral replication and reservoirs in peripheral blood, as measured by cell-associated HIV-1 RNA and DNA, respectively, were demonstrated to be similarly low in both cohorts. These two virologically matched cohorts were then comprehensively analysed by polychromatic flow cytometry for HIV-1-specific CD4^+^ and CD8^+^ T-cell functional profile in terms of cytokine production and cytotoxic capacity using IFN-γ, IL-2, TNF-α production and perforin expression, respectively. Comparable levels of highly polyfunctional HIV-1-specific CD4^+^ and CD8^+^ T-cells were found in LTTS and LTNPs, with low perforin expression on HIV-1-specific CD8^+^ T-cells, consistent with a polyfunctional/non-cytotoxic profile in a context of low viral burden.

**Conclusions:**

Our results indicate that prolonged ART initiated at the time of HIV-1 seroconversion is associated with immuno-virological features which resemble those of LTNPs, strengthening the recent emphasis on the positive impact of early treatment initiation and paving the way for further interventions to promote virological control after treatment interruption.

## Introduction

Antiretroviral therapy (ART) is considered life-long once initiated at the time of chronic HIV-1 infection. It remains unclear whether we can change the course of the disease, decrease long-term exposure to medication and promote control of viremia after discontinuation of treatment initiated at the time of seroconversion [1]. Residual reservoirs in long-lived CD4^+^ T-cells, in particular, are understood to be responsible for the virological rebound observed after treatment interruption [2], [3]. Previous studies in early infection using various treatment durations have shown variable degrees of immune preservation and decrease of the saturation of peripheral viral reservoirs overtime. We have therefore hypothesized that a very prolonged treatment period initiated at seroconversion might allow the preservation/reconstitution of HIV-1-specific immunity as well as a substantial decline in viral burden and promote a non-progressive type of infection. Therefore, in order to help clarify this issue, we have used in this study as a comparator to long-term treated seroconverters (LTTS) a population of long-term non-progressors (LTNPs) which remains disease-free and spontaneously controls CD4^+^ T-cell loss and viral replication in the absence of treatment.

We have concentrated our analysis on HIV-1-specific T-cells which have been shown to play a substantial role in HIV-1 infection control. Previous studies have demonstrated an absence of correlation between the magnitude of interferon-gamma (IFN-γ) production and viremia levels. As a result the emphasis has been recently put on their qualitative rather than quantitative features [4], [5]. The term “polyfunctional” is therefore commonly used to define a type of T-cell immune responses that, in addition to typical effector functions such as cytotoxic activity and secretion of IFN-γ or tumor necrosis-alpha (TNF-α), also comprises distinct T-cell populations able to secrete interleukin-2 (IL-2) and to retain proliferative capacity [5], [6], [7], [8]. A polyfunctional CD8^+^ T-cell profile has been associated with protective antiviral immunity in several viral infections, including HIV-1 [6], [9], [10], [11]. Cytotoxicity is a further function by which CD8^+^ T-cells mediate anti-tumor and anti-viral activity [12], [13], [14] and we have recently shown that perforin expression is the most powerful correlate of cytotoxic function in human viral infections [15].

Studies have indeed demonstrated that HIV-1-specific CD8^+^ T-cells in LTNPs are functionally fit in terms of cytokine production as well as proliferative and cytotoxic capacity, in contrast to what is observed in chronic progressors [9], [16], [17], [18], [19], [20]. Slow and incomplete improvement of HIV-1-specific CD8^+^ T-cell functionality in progressors has been noted when ART is initiated during chronic infection, even when maintained for prolonged periods [19], [21]. As a rule treatment interruption has been associated with viral rebound which suggests that residual functional immunological defects are still present after many years of treatment-induced aviremia in chronically infected patients [21]. Recent studies have indeed shown that polyfunctionality, proliferation and cytotoxicity capacities are not recovered to the same extent as in LTNPs when ART is initiated during the chronic stage of the infection [19], [22]. However, preservation and enhanced reconstitution of these immune functions as well as a substantial decrease of viral reservoirs may occur with an early and prolonged treatment intervention able to produce at the time of acute infection a rapid viremia decline and subsequent prolonged aviremia.

In this study we have compared HIV-1-specific CD4^+^ and CD8^+^ T-cell functionality, immune activation, HIV-1 reservoirs and residual replication in peripheral blood of LTTS with those of LTNPs. We have measured cell-associated HIV-1 DNA and RNA levels to quantify reservoirs and residual viral replication, respectively and assessed CD8^+^ T-cell immune activation by CD38^+^ expression. HIV-1-specific CD4^+^ and CD8^+^ T-cell responses (initially screened by IFN-γ ELISpot assay) were analysed by polychromatic intracellular cytokine staining (ICS) in order to assess polyfunctionality as defined by the production of multiple cytokines (IFN-γ, IL-2 and TNF-α) and the ex-vivo perforin expression by HIV-1-specific CD8^+^ T-cells.

Interestingly enough, similarly low levels of cell-associated HIV-1 DNA and RNA as well as CD8^+^/CD38^+^ T-cells were detected in peripheral blood in the two subject groups. We have also demonstrated the presence of highly polyfunctional HIV-1-specific CD4^+^ and CD8^+^ T-cells in all LTTS and LTNPs and little perforin expression on HIV-1-specific CD8^+^ T-cells in both cohorts.

Our study design allowed overcoming the recurrent bias of the different viral burden present in previous studies which have compared HIV-1 with other persistent human viral infections (e.g. CMV, EBV vs. HIV) or cohorts of HIV-1 progressors vs. non-progressors for immune correlates of viral control. We had in contrast the unique opportunity to compare two cohorts of HIV-1-infected subjects with a strikingly similar viral burden. This is of relevance since immune correlates of virological control are known to be associated with the extent of viral burden [5].

Our results therefore raise the possibility of HIV-1-specific T-cell immune preservation/reconstitution with early and prolonged ART in a context of low viral burden in peripheral blood and provide a detailed comparison of HIV-1-specific T-cell profile in subjects with similar viral burden in ART-induced and spontaneous control of viral replication. These data should guide further therapeutic studies in HIV-1 seroconverters to investigate newer therapeutic compounds aimed at further decreasing viral reservoirs and test the possibility of long-term control of viremia after treatment discontinuation [23]. The comprehensive information in terms of HIV-1-specific T-cell functional profile and viral reservoirs in LTNPs provided here should also increase our understanding of correlates of viral control and help contribute to vaccine development.

## Methods

### Study population

The study included a total of 35 patients who were prospectively enrolled into 2 cohorts between March 2007 and March 2008. Their characteristics are described in Table 1.

**Table 1:** Subjects Characteristics.

	LTTS^A^ (n = 20)	LTNPs^A^ (n = 15)	P value (α = 0.003)
**Males** (%)	19 (95)	11 (73)	0.14
**Age** * (range)	40 (29–59)	41 (27–67)	0.5
**Caucasian** (%)	19 (95)	14 (93)	1
**MSM** B (%)	18 (90)	8 (53)	0.02 (N.S.)^C^
**HTS** B (%)	2 (10)	5 (33)	0.11
**HAEM** B (%)	0 (0)	2 (13)	0.18
**Years of infection** * (range)	6 (4–7)	13 (7–25)	n.a.^D^
**CD4^+^ T-cells** * (cells/µL; range)	800 (567–1412)	783 (433–1648)	0.29
**CD4^+^/CD8^+^ T-cell ratio** * (range)	1.1 (0.65–3.70)	1.2 (0.31–1.90)	0.06
**CD8^+^/CD38^+^ T-cells** * (x10^9^cells/L; range)	0.05 (0.008–0.274)	0.06 (0.016–0.534)	0.23
**CD8^+^/CD38^+^ T-cells** * (%; range)	7 (3–25)	6 (3–30)	0.28
**pVL** E * (HIV-1 RNA copies/mL)	all <50	11 <50, 4 <1000	n.a.^D^
**Cell-associated HIV-1 RNA** * (copies/10^6^ PBMCs; range)	3.9 (0–36)	5.8 (0–10.3)	0.16
**Cell-associated HIV-1 DNA** * (copies/10^6^ PBMCs; range)	47.7 (4.8–583.2)	19.7 (0.5–295.5)	0.10
Patients with **HLA-B** * **5701** allele (%)	1 (5)	4 (27)	0.14
Patients with **HLA-B** * **5701, -B** * **2705, -B** * **5801, -B** * **5101, -B** * **1302** alleles (%)	5 (25)	6 (40)	0.45
Patients with **HLA-B** * **3503, -B** * **5301, -B** * **1801** alleles (%)	3 (15)	2 (13)	1

A LTTS, long-term treated HIV-1 seroconverters; LTNPs: HIV-1 long-term non-progressors.

B MSM, men-having-sex-with-men; HTS, heterosexuals; HAEM, haemophiliacs.

C N.S., not statistically significant after Bonferroni correction for multiple testing (the cut-off for statistical significance is 0.003).

D n.a, not applicable.

E pVL, plasma viral load.

*Median values at time of sampling.

The first cohort included 20 long-term ART-treated seroconverters (LTTS) who had chosen to initiate treatment at the time of HIV-1 seroconversion and had been followed up in the same treatment centre since diagnosis. They were selected from the Royal Free Hospital (RFH) cohort of seroconverters at the Ian Charleson Day Care Centre (ICDC) using our HIV database on the basis of the following inclusion criteria: (a) ART for at least 4 years with no treatment interruption; (b) long-term aviremia (<50 HIV-1 RNA copies/mL) and (c) an absence of treatment failure defined by a viral load above 400 HIV-1 RNA copies/mL. Seroconversion to HIV-1 was defined by: (a) negative HIV-1 antibody by ELISA and evidence of HIV-1 viremia ≥5,000 HIV-1 RNA copies/mL plasma and/or (b) incomplete HIV Western Blot with ≤3 bands and/or detuned assay with a value of <0.6 for clade B patients. At the time of seroconversion, median CD4^+^ T-cell count was 474 (range: 187–1033) and plasma viral load 750.000 HIV-1 RNA copies/mL (range: 38.300–7.500.000). Duration of ART was calculated from the day of initiation of ART until the time of sampling. Treatment (2 non-nucleoside analogues inhibitors of the HIV-1 reverse transcriptase and 1 boosted inhibitor of HIV-1 protease) was initiated shortly after the diagnosis of HIV was made (median time from diagnosis to treatment initiation: 13 days).

The second cohort included 15 long-term non-progressor subjects (LTNPs) recruited at the Ian Charleson Day Centre (ICDC) (n = 9) and Haemophilia Centre (n = 2) at the RFH in London using the centre’s HIV database as a reference, and referrals from University College Hospital, London (n = 1), North Middlesex Hospital, London (n = 1), University Hospital of Wales, Cardiff (n = 1) and at Victoria Hospital, Blackpool (n = 1) in the UK. Inclusion criteria were: (a) ≥7 years of documented HIV-1 infection; (b) viremia <1000 HIV-1 RNA copies/mL plasma in >90% of measurements; (c) ≥500 CD4^+^ T-cells/mm^3^ in ≥90% of measurements; (d) absence of AIDS-defining conditions; (e) ART-naive except for zidovudine use for the prevention of mother-to-child transmission (n = 2). Patients’ medical files were reviewed in order to confirm inclusion criteria. Patient characteristics are described on Table 1.

As shown in Table 1, the 2 cohorts were matched for major clinical and laboratory parameters. Men who had sex with men were in a majority in both cohorts although females were better represented in LTNPs. Both cohorts were similar in terms of HIV-1 viral load and CD4^+^ T-cell counts at the time of blood sampling. Median CD4^+^ T-cell count was 783 (range: 433–1648) and 800 (range: 567–1412) for LTNPs and LTTS, respectively (P = 0.29). All LTTS and LTNP subjects were below 50 HIV-1 RNA copies/ml at the time of sampling except for 4 LTNPs (381, 240, 636, 130 HIV-1 RNA copies/mL). Therefore, the vast majority of LTNPs also fulfilled the criteria for elite controllers [24]. Median duration of infection at the time of sampling was 13 years (range: 7–25) for LTNPs and 6 years (range: 4–7) for LTTS.

### Ethics Statement

Informed written consent was obtained from all subjects. The study was approved by the Royal Free Hospital and NHS ethics committees and the Institutional Review Board of the Centre Hospitalier Universitaire Vaudois.

### Blood sampling and PBMC isolation and storage

A single blood sample was performed at the ICDC at the RFH in London for research purposes. Processing of EDTA samples for virological and immunological analysis was performed at the Department of Immunology at the RFH within 3 hours of venopuncture. Peripheral blood mononuclear cells (PBMCs) were isolated by Ficoll-Paque density gradient centrifugation from heparinized blood and stored in 7.5% DMSO in liquid nitrogen.

We also performed on the day of blood sampling routine full blood count, biochemistry, hepatitis B and C serology, CD4^+^ and CD8^+^ T-cell counts, CD4^+^/CD8^+^ T-cell count ratio, CD8^+^/CD38^+^ T-cell absolute counts and percentages, HIV-1 viremia and viral clade and HLA class I determination. Levels of CD8^+^/CD38^+^ T-cells were quantitated according to a previously published method [25]. HIV-1 pol gene sequences for codons 1–99 of the protease and 1–335 of reverse transcriptase (RT) were obtained from whole EDTA blood samples using the Vircoseq^TM^ HIV-1 genotyping system (Abbott Laboratories/Celera Diagnostics, USA) according to the manufacturer’s instructions. Sequences were analysed with a 3100-Avant Genetic Analyzer (Applied Biosystems, UK) and edited using Seqscape v2.5 (Applied Biosystems, UK). Subtypes were assigned by phylogenetic analysis using the software MEGA v4 [26] and by comparing to known reference HIV-1 sequences derived from the Los Alamos database [27].

### HLA class I genotyping

High-resolution sequence-based typing was carried out for HLA-A and HLA-B, as previously described [28]. Briefly, generic PCR co-amplification of both maternal and paternal alleles was carried out to create PCR products specific for these loci. Cycle sequencing using the Dye terminator method was then carried out using ABI Prism® BigDye® Terminator v3.1 chemistry. Sequencing was targeted at exons 2, 3 and 4. The results generated were compared with previous Luminex® typing to remove any ambiguous combinations not seen by this technique.

HLA-C genotyping was performed at an intermediate resolution level using LABType® SSO Typing Test according to the manufacturer’s instructions. Data were analysed on a Luminex® 100 flow analyser.

### Synthetic peptides used in IFN-γ ELISpot and polychromatic flow cytometry assay (ICS)

All peptides used in this study were HPLC purified (>80% purity). We selected 191 HIV-1-derived epitopes (9/10-mers) covering different regions (Gag, Env, Pol, Nef, Tat, Rev, Vpr, Vpu and Vif) of HIV-1 from consensus strain IIIB. Only optimal CD8^+^ T-cell epitopes with known HLA class I restriction were selected [29]. Fine epitope mapping was thus performed on the predicted HLA class I genotype according to Los Alamos database and patient HLA class I genotype. The 191 optimal CD8^+^ T-cell epitopes were tested individually in the polychromatic intracellular cytokine staining (ICS) assay and organized into 28 pools of multiple peptides based on a 14×14 matrix such that each epitope was present in precisely 2 pools [30]. Furthermore, a pool of 105 peptides (15-mers overlapping by 11 amino acids (aa) encompassing the Gag protein (aa *gag* 1 - *gag* 432) of HIV-1 from consensus strain IIIB was used in ICS in order to evaluate the response to this protein by the CD4^+^ T-cell subset. In addition, a pool of peptides (n = 23) most frequently recognized in Cytomegalovirus, Epstein Barr Virus and Flu (pool CEF) infections was used to assess the functional profile of non-HIV-specific CD8^+^ T-cell responses [31].

### Interferon-gamma (IFN-γ) enzyme-linked immunospot (ELISpot) assay

Initial screening for HIV-1-specific CD8^+^ T-cell responses in the 2 study cohorts was performed by IFN-γ ELISpot assay. The assay was conducted on cryopreserved peripheral blood mononuclear cells (PBMCs) using Becton Dickinson (BD, San Diego, USA) human IFN-γ ELISpot kit according to the provided protocol. Briefly, PBMCs were plated at 10^6^ cells/well after 4–6 hour resting. Stimulation was performed in triplicates in the presence of 1 µg/well of peptide (from the panel of 191 HIV-1 selected peptides organized in a matrix pool) or 200 ng/well of staphylococcal enterotoxin B (SEB) used as a positive control. In addition, a negative control with medium alone was always included in each test. Plates were stored at 37°C in a 5% CO_2_ incubator overnight, washed, and coated with detection antibody for 2 hours at room temperature. They were again washed and coated with avidin-peroxidase for 1 hour at room temperature. Plates were then washed and developed by addition of AEC (3-Amino-9-ethyl-carbazole) substrate. Responses were expressed as net spot-forming units (SFU) per 10^6^ PBMCs. The following criteria were used to define the technical validity of the assay and positive responses: (a) The number of spot forming units (SFU)/10^6^ PBMCs in the assay background (unstimulated PBMCs) had to be <50 and the positive control responses to SEB >500 SFU/10^6^ PBMCs; (b) Responses were considered positive when ≥fourfold above background and ≥55 SFU/10^6^ PBMCs. HIV-1-specific T-cell responses were further characterized using polychromatic intracellular cytokine staining (ICS) in responders with IFN-γ ELISpot responses in the range of 100 SFU/10^6^ cells or above.

### Antibodies (Abs)

The following Abs were used in the ICS assay: CD8-PB, CD4-ECD, IFN-γ-APC, TNF-α-PECy7 and IL-2-PE (BD, San Diego, USA), perforin-FITC (Biolegend, San Diego, USA) and CD3-APCCy7 (Invitrogen, Carlsbad, USA).

### Polychromatic intracellular cytokine staining (ICS)

Cryopreserved PBMCs were thawed and rested overnight in R10 media (RPMI Glutamax-1 containing 10% heat-inactivated fetal calf serum) at a concentration of 10^6^ cells/mL. The following day 1–2×10^6^ cells were stimulated for 6 h in 1 ml of R10 media in the presence of Golgiplug [1 µl/ml, Becton Dickinson (BD), San Diego, CA], purified soluble anti-CD28 Ab (0.5 µg/ml, BD San Diego, CA) and 1 µg/ml of peptide [32]. An unstimulated (R10 only) and positive control (SEB 200 ng/ml) were included in each assay. At the end of the stimulation period, cells were washed, stained for dead cells using the Aqua LIVE/DEAD stain kit (Invitrogen), permeabilized (Cytofix/Cytoperm, BD) and then stained at RT for 30’ with CD3, CD4, CD8, IFN-γ, TNF-α, IL-2 and, when indicated, perforin. Cells were then fixed and acquired on an LSR SORP flow cytometer (BD). Data were analysed using FlowJo software (version 8.8.2; Tree Star). For the analysis of polyfunctionality, the FlowJo Boolean Gate Platform was used to create 8 and 16 patterns of responses for the 3 or 4 tested functions, respectively. The gating scheme used for all ICS assays performed is shown in Data S1. Data were plotted and further analysed with SPICE software (version 4.2.3; from M. Roederer, National Institutes of Health, Vaccine Research Center, Bethesda, MD). All reported values have been corrected for background. The number of lymphocyte-gated events ranged between 6×10^5^ and 1×10^6^ in the flow cytometry experiments. The mean of the background in the unstimulated controls +2 standard deviations never exceeded 0.05%. An ICS result to be considered positive had display more than 0.05% of cytokine-positive cells and to be ≥threefold above background.

### Virological studies

Cell-associated HIV-1 RNA and DNA levels were quantitated using a previously published method [33] with a limit of detection of 3 copies/10^6^ PBMCs. We have reported levels of copies per million PBMCs as in other studies and not per CD4^+^ T-cells as CD4^+^ T-cell counts in both cohorts were high and there was no predicted dilution effect in the absence of variation in CD4^+^ T-cell counts.

### Statistical analysis

Statistical significance (P values) of the results was calculated by two tails student t-test using either Excel (Microsoft, Redmond, WA) or SPICE 4.2.3. Correlations among variables were tested by simple regression analysis. Proportions were compared with Fisher’s exact test using GraphPad Prism version 3.0 (GraphPad Software, San Diego, CA, USA). The level of significance was set at P<0.05 and Bonferroni adjustment of P-values was applied in case of multiple testing.

## Results

### HLA class I analysis in LTTS and LTNPs

Several lines of evidence have stressed in recent years the importance of HLA class I genotype in predicting progression [34], [35], [36] with certain alleles associated with a slower course of the infection. Therefore, in order to exclude large differences in the genetic background of our 2 cohorts, we assessed the HLA class I genotype in all study patients. High- and intermediate-resolution HLA class I typing was performed as described in the method section for loci A and B, and for locus C, respectively. As shown in Table 1, when evaluating the frequency of HLA alleles known to be associated with slow (HLA-B*5701/02/03, *2705, *5801, *5101, *1302) or rapid (HLA-B*5802, *3502/03, *5301, *1801) disease progression [37], we found a trend towards a higher frequency of HLA-B*5701 alleles in LTNPs as compared to LTTS (27% of LTNPs vs. 5% of LTTS were B*5701^+^; P = 0.14) which confirmed the previously described enrichment of this allele in LTNPs and showed that this was the only major difference in terms of HLA class I genotype between the two cohorts studied.

### Similarly low levels of HIV-1 reservoirs, residual replication and CD8^+^/CD38^+^ T-cells in LTTS and LTNPs peripheral blood

Residual viral replication and persistence of HIV-1 in long-lived reservoirs in resting CD4^+^ T-cells are associated with viral rebound upon treatment cessation and represent major obstacles to eradication [3], [38], [39], [40], [41], [42]. Cell-associated HIV-1 RNA and DNA have been detected in the resting CD4^+^ T-cell compartment in the peripheral blood of the majority of infected individuals with undetectable viremia while on ART [43]. Their levels have been used in several previous studies to quantify the saturation of viral reservoirs and residual viral replication in peripheral blood in aviremic subjects, respectively. Cell-associated HIV-1 DNA levels have been correlated to the extent of virological rebound after ART discontinuation in subjects with treatment initiation during acute or chronic infection [44], [45]. Recent studies have suggested that early treatment initiation may accelerate HIV-1 decay both in blood and gut-associated lymphoid tissue [42], [46], [47]. Therefore, in order to better quantify viral burden in our two cohorts, we have used these two further virological markers in addition to plasma viremia.

When comparing cell-associated HIV-1 DNA and RNA between the two cohorts, we found that all study subjects had similarly low levels. Results are shown in Table 1. The median number of copies of cell-associated HIV-1 DNA per 10^6^ PBMCs was 47.7 (range 583.2–4.8) and 19.7 (range 295.5–0.5) in LTTS and LTNPs, respectively (P = 0.10). Median HIV-1 RNA levels were 3.9 (range 45.8–0.7) and 5.8 copies per 10^6^ PBMCs (range 10.3–1.4) in LTTS and LTNPs (P = 0.16), respectively. Altogether these results show strong similarities between the two cohorts using virological markers aimed at further assessing viral reservoirs and residual replication in peripheral blood in these aviremic subjects.

T-cell immune activation is known to play an important role in HIV pathogenesis and is linked to CD4^+^ T-cell decline and disease progression [48], [49]. Although CD38 can also be expressed on the surface of thymocytes and naïve T-cells, it is widely used as an activation marker in HIV infection. We have previously described that early initiation of ART at seroconversion is associated with decreasing levels of CD8^+^/CD38^+^ T-cells [25]. The expression of CD38 on HIV-1-specific CD8^+^ T-cells has been shown to be low in LTNP cohorts and similar to that found in successfully treated patients [48], [50]. However, comparative data between LTTS and LTNPs for CD8^+^/CD38^+^ T-cells are not available and this marker could be an indicator of the extent of immune reconstitution which is taking place with prolonged ART initiated at seroconversion.

In this study, we were able to show further similarities between the two cohorts in terms of CD8^+^ T-cell activation as measured by CD38 expression. Both displayed normal levels of CD8^+^/CD38^+^ T-cells (normal range for absolute count: 0.2–0.8×10^9^/L and percentage: 3–22% of lymphocytes). Median absolute CD8^+^/CD38^+^ T-cell counts and percentages were 0.06 and 0.05×10^9^/L (P = 0.23) and 6% and 7% (P = 0.28) for LTNPs and LTTS, respectively.

### Similar magnitude and functional profile of HIV-1 Gag-specific CD4^+^ T-cell responses in LTTS and LTNPs

We wanted to investigate in details the effect of prolonged treatment started in early infection on HIV-1-specific CD4^+^ T-cells as these cells play a central role in sustaining CD8^+^ T-cell functions. It has been previously demonstrated that HIV-1-specific CD4^+^ T-cell functional profile is driven by the level of antigen exposure: the cytokine producing pattern is skewed towards IFN-γ only production in viremic individuals but can be rescued by ART intervention [51], [52].

**Figure 1:**
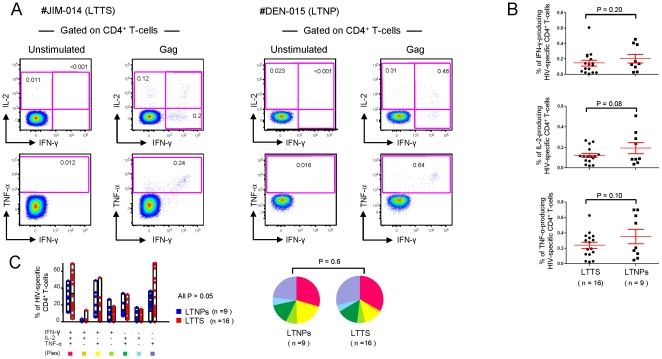
Functional cytokine profile of HIV-1 Gag-specific CD4^+^ T-cells. (**A**) Shown are two representative flow cytometry profiles of Gag-specific CD4^+^ T-cell responses from a LTTS (dot plots on the left) and a LTNP (dot plots on the right) subject. The production of IL-2, TNF-α and IFN-γ was measured upon 6 hours of *in vitro* stimulation with the Gag peptide pool. (**B**) Cumulative data (mean±SE) on the percentage of IFN-γ-, IL-2- and TNF-α-producing HIV-1 specific CD4^+^ T-cells following stimulation with the Gag peptide pool. (**C**) Cumulative data of the simultaneous analysis of IFN-γ, IL-2 and TNF-α production. All possible combinations of IFN-γ, IL-2 and TNF-α production are shown on the x axis, whereas the percentage of the various cytokine-producing cell subsets within HIV-specific CD4^+^ T-cells is shown on the y axis. Pie charts summarize the data, and each slice corresponds to the proportion of virus-specific CD4^+^ T-cells positive for a given combination of T-cell functions. LTTS: long-term treated HIV-1 seroconverters; LTNPs: HIV-1 long-term non-progressors.

It was thus important to further characterize HIV-1-specific CD4^+^ T-cell functional profile in a seroconverter cohort with long-term treatment and to compare it with LTNPs. Moreover, our LTTS and LTNP cohorts were shown to be highly homogenous for low viral burden thus providing the opportunity to compare the HIV-1-specific CD4^+^ T-cell functional profile in conditions of ART-induced vs. spontaneous controlled viremia. HIV-1-specific CD4^+^ T-cells were therefore characterized in a randomly selected subgroup of 25 subjects (16 LTTS and 9 LTNPs) for IFN-γ, TNF-α and IL-2 production upon stimulation with the Gag peptide pool. A representative example of results for each cohort is shown in Figure 1A.

We demonstrated that Gag-specific CD4^+^ T-cell responses were detected in all subjects. When quantifying the amount of IFN-γ, TNF-α and IL-2-producing cells upon stimulation with the Gag peptide pool, LTTS and LTNPs showed overall similar levels as shown in Figure 1B. After subtraction of the background, the total cytokine response was 0.28% and 0.33% of CD4^+^ T-cells in LTTS and LTNPs, respectively (P = 0.28). The mean percentage of Gag-specific CD4^+^ T-cells producing IFN-γ was 0.15% (range 0.01%–0.61%) and 0.21% (range 0.03%–0.46%) in LTTS and LTNPs, respectively (P = 0.20). Values in LTTS and LTNPs for TNF-α were 0.24% (range 0.02%–0.63%) and 0.35% (range 0.05%–0.70%) (P = 0.10) and for IL-2 0.12% (range 0.02%–0.27%) and 0.19% (range 0.04%–0.51%) (P = 0.08).

We next analysed the Gag-specific CD4^+^ T-cell functional profile based on its ability to secrete one or more cytokines. As shown in Figure 1C, a similar polyfunctional profile, as defined by the presence of HIV-1-specific CD4^+^ T-cells producing simultaneously more than one cytokine was present. Polyfunctional CD4^+^ T-cell populations accounted for more than 50% of the total response in both cohorts (61% in LTTS and 64% in LTNPs; P = 0.39). In particular, the mean percentage of the ‘triple positive’ population, i.e. the cells producing simultaneously IFN-γ, IL-2 and TNF-α, was 30% (range 11%–49%) and 33% (range 3%-69%) in LTTS and LTNPs, respectively (P = 0.32).

We were therefore able to demonstrate robust and polyfunctional HIV-1-Gag-specific CD4^+^ T-cell responses of similar intensity and functional profile in both cohorts.

### Higher breadth and magnitude of HIV-1-specific CD8^+^ T-cell responses in LTNPs as compared to LTTS

Robust HIV-1-specific CD8^+^ T-cells have been described in LTNPs in contrast to those found in aviremic patients receiving effective ART during chronic infection [18], [22], [53], [54]. In order to clarify whether early treatment initiation could lead to a different pattern of responses than the one found in treated chronic infection, we initially screened our subjects for HIV-1-specific CD8^+^ T-cell responses by IFN-γ ELISpot assay using the epitope matrix pool described in the method section. High levels of IFN-γ-producing T-cells were detected in all subjects but the breadth and magnitude of responses, however, differed between the two cohorts. A higher mean number of responding pools (14 versus 8; P = 0.0005) and SFU/10^6^ PBMCs (665 versus 426; P = 0.00001) was found in LTNPs as compared to LTTS.

**Figure 2:**
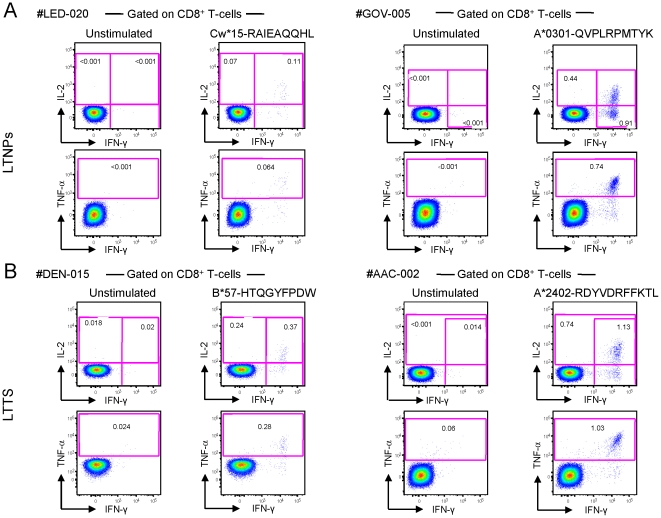
Flow cytometry profiles of HIV-1-specific CD8^+^ T-cells. (A ) Shown are two representative flow cytometry profiles of HIV-1-specific CD8^+^ T-cell responses from two LTTS subjects: left panels from subject #LED-020 (stimulation with a gp41 epitope, aa 46–54) and right panels from subject #GOV-005 (stimulation with a nef epitope, aa 73–82). (**B**) Shown are two representative flow cytometry profiles of HIV-1-specific CD8^+^ T-cell responses from two LTNP subjects: left panels from #DEN-015 (stimulation with a nef epitope, aa 116–124) and right panels from #AAC-002 (stimulation with a p24 epitope, aa 162–172). IL-2, TNF-α and IFN-γ production was measured upon 6 hours of *in vitro* stimulation with optimal HIV-1 peptides. LTTS: long-term treated HIV-1 seroconverters; LTNPs: HIV-1 long-term non-progressors.

We then performed fine epitope mapping by matching subjects’ HLA class I genotype with ELISpot assay results according to the Los Alamos database of HLA class I restriction. HIV-1-specific T-cell responses were further characterized in patients with IFN-γ ELISpot responses in the range of 100 SFU/10^6^ cells or above by ICS analysis for IFN-γ, IL-2 and TNF-α production upon stimulation with the optimal mapped CD8^+^ T-cell peptides. Thus, 103 epitope-specific CD8^+^ T-cell responses (63 from LTNPs and 40 from LTTS) were analysed by ICS. Representative examples of these responses are shown in Figure 2A and B.

In line with the ELISpot data, we found a higher breadth of responses in LTNPs by ICS analysis with LTTS and LTNPs subjects targeting a median of 2 (range 1–5) and 5 (range 1–9) epitopes, respectively (P = 0.001). These results were still significant when HLA-B*5701^+^ subjects were removed from the analysis, indicating that the higher number of targeted epitopes in LTNPs was independent of the HLA-B*5701 imbalance between the 2 cohorts.

**Figure 3:**
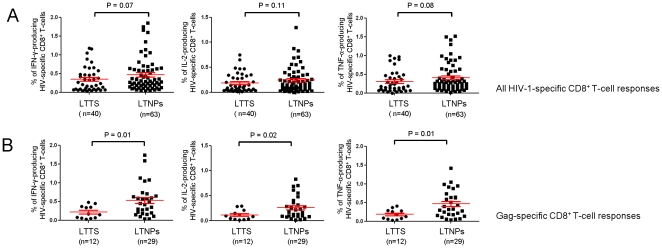
Comparison of the magnitude of HIV-1-specific CD8^+^ T-cell responses between LTTS and LTNPs. Cumulative data (mean±SE) of the percentage of IFN-γ-, IL-2- and TNF-α-producing HIV-1 specific CD8^+^ T-cells following 6 hours of *in vitro* stimulation with optimal CD8^+^ T-cell HIV-1 peptides (A) or with optimal CD8^+^ T-cell Gag-derived peptides (B). LTTS: long-term treated HIV-1 seroconverters; LTNPs: HIV-1 long-term non-progressors.

Alongside the higher breadth, the increased magnitude of responses found in LTNPs by ELISpot assay was confirmed by ICS analysis, although not reaching statistical significance, when we determined the percentage of IFN-γ-, TNF-α- and IL-2-producing CD8^+^ T-cells following stimulation with specific peptides (Figure 3A). After subtraction of the background, the total cytokine response was 0.37% and 0.50% of HIV-1 specific CD8^+^ T-cells in LTTS and LTNPs, respectively (P = 0.06). The mean percentage of epitope-specific IFN-γ-producing CD8^+^ T-cells was 0.34% (range 0.03%–1.18%) and 0.47% (range 0.04%–1.85%) in LTTS and LTNPs, respectively (P = 0.07). Respective values in LTTS and LTNPs for TNF-α production were 0.31% (range 0%–1%) and 0.40% (range 0.02%–1.51%) (P = 0.08) and for IL-2 production 0.19% (range 0.01%–0.75%) and 0.24% (range 0%–1.30%) (P = 0.11).

We next determined whether HIV-1-specific CD8^+^ T-cell responses targeting viral proteins or epitopes shown to be associated with viral control (“favourable epitopes”) [55], [56], [57] and those restricted by the HLA-B*5701 allele showed different levels of cytokine production. Gag responses were shown to contribute substantially to the higher magnitude of responses found in LTNPs. As shown in Figure 3B, IFN-γ, TNF-α and IL-2 production by CD8^+^ T-cells upon stimulation with Gag peptides was significantly higher in LTNPs than in LTTS. After subtraction of the background, the mean percentage of Gag-specific CD8+ T-cells producing IFN-γ was 0.22% and 0.54% in LTTS and LTNPs, respectively (P = 0.01). Respective values in the LTTS and LTNP groups for TNF-α were 0.18% and 0.46% (P = 0.01) and for IL-2 0.11% and 0.26% (P = 0.02). In contrast, CD8^+^ T-cell responses targeting proteins other than Gag were of similar intensity in both cohorts (data not shown). When comparing between the two cohorts responses targeting “favourable epitopes” (Data S2 A) and HLA-B*5701-restricted responses (Data S2 B), we found similar levels of IFN-γ, TNF-α and IL-2 production which matched those observed for CD8^+^ T-cell responses targeting epitopes other than the “favourable” ones and which were restricted by HLA class I alleles other than HLA-B*5701 (data not shown). However, these analyses were limited by the small number of responses targeting “favourable epitopes” (n = 2) and HLA-B*5701-restricted responses (n = 2) in LTTS. In addition, we found that the majority of “favourable epitope”-targeting (5 out of 7) and HLA-B*5701-restricted responses (7 out of 13) in LTNPs were directed towards the Gag region and were among the strongest responses that we could observe.

In line with a recent report [58], the trend for stronger HIV-1-specific CD8^+^ T-cell responses and lower cell-associated HIV-1 DNA in LTNPs as compared to LTTS resulted in a higher ratio of these 2 parameters in LTNPs (0.45 vs. 0.03; P = 0.02). However, the statistically significant difference was lost when we analysed the ratio of Gag-specific CD8^+^ T-cells and cell-associated HIV-1 DNA (0.33 vs. 0.02; P = 0.06).

Of interest, we found that the trend towards a lower magnitude of CD8^+^ T-cell responses observed in LTTS was HIV-1 specific. In a subgroup of 19 subjects (12 LTTS and 7 LTNPs) for whom suitable numbers of PBMCs were available, we characterized CD8^+^ T-cell responses based on IFN-γ, TNF-α and IL-2 production upon stimulation with CMV, EBV and Flu peptides (CEF pool). Our results showed that LTTS displayed stronger CD8^+^ T-cell responses than LTNPs (data not shown).

Overall, these data show that the breadth and magnitude of HIV-1-specific CD8^+^ T-cell responses differed between the two groups. Although LTTS showed strong virus-specific CD8^+^ T-cell responses, these were more robust and diverse in LTNPs.

### Frequency of distinct optimal HIV-1-specific CD8^+^ T-cell epitope recognition

We have shown a wider breadth of HIV-1-specific CD8^+^ T-cell responses in LTNPs and next investigated whether this was matched by a different frequency of epitope-targeting.

We first assessed whether viral proteins were targeted with similar frequencies in the two cohorts and found a trend towards LTNPs targeting more Gag and RT-Pol epitopes and LTTS targeting more Env epitopes. However, the frequencies of recognition were too low to allow solid comparisons between the two groups.

We next evaluated the frequency of “favourable epitopes” targeted among all recognized HIV-1-specific CD8^+^ T-cell epitopes in both cohorts. Four “favourable epitopes” were included in the panel of 191 optimal CD8^+^ T-cell epitopes used in the ICS assay: KK10-B*2705, TW10-B*5701, HW9-B*5701 and DA9-B*1402. We found that these epitopes were among the most commonly recognized (≥40%) in both cohorts. The frequency of recognition was 67%, 60%, 40% and 100% for KK10-B*2705, TW10-B*5701, HW9-B*5701 and DA9-B*1402, respectively. Moreover, LTNPs recognized more frequently the “favourable epitopes” than LTTS (13% vs. 5%; P = 0.24). This difference was mainly explained by the higher frequency of the HLA-B*5701 allele in LTNPs since 50% (2 out of 4) of the “favourable epitopes” tested in the ICS assay were HLA-B*5701-restricted. We could also confirm [35] that epitopes restricted by HLA-B alleles were more frequently recognized than those restricted by HLA-A (P = 0.005) or -C alleles (P = 0.066).

We observed that in HLA-B*5701^+^ patients HIV-1-specific CD8^+^ T-cell responses were largely focused on epitopes restricted by this allele. Among the 5 HLA-B*5701^+^ patients (4 LTNPs and 1 LTTS), 3 had 100%, 1 had 80% and 1 had 60% of CD8^+^ T-cell responses targeting epitopes restricted by this allele. These data confirm previous reports [36], [59] of the preferential targeting of HLA-B*5701 restricted epitopes in patients positive for this allele.

### Similar levels of polyfunctional HIV-1-specific CD8^+^ T-cell responses in both cohorts

A growing body of evidence indicates that the independent assessment of single functions may not be particularly informative, whereas the simultaneous analysis of multiple parameters can provide a more accurate picture of CD8^+^ T-cell functionality. In particular, polyfunctional CD8^+^ T-cells (i.e. CD8^+^ T-cells exhibiting simultaneously multiple functions) have been associated with protective antiviral immunity and the control of viral infections [6], [9], [10], [11]. In the present study we had the unique opportunity to compare the level of HIV-1 specific CD8^+^ T-cell polyfunctionality between these two cohorts which were matched for viral burden.

**Figure 4:**
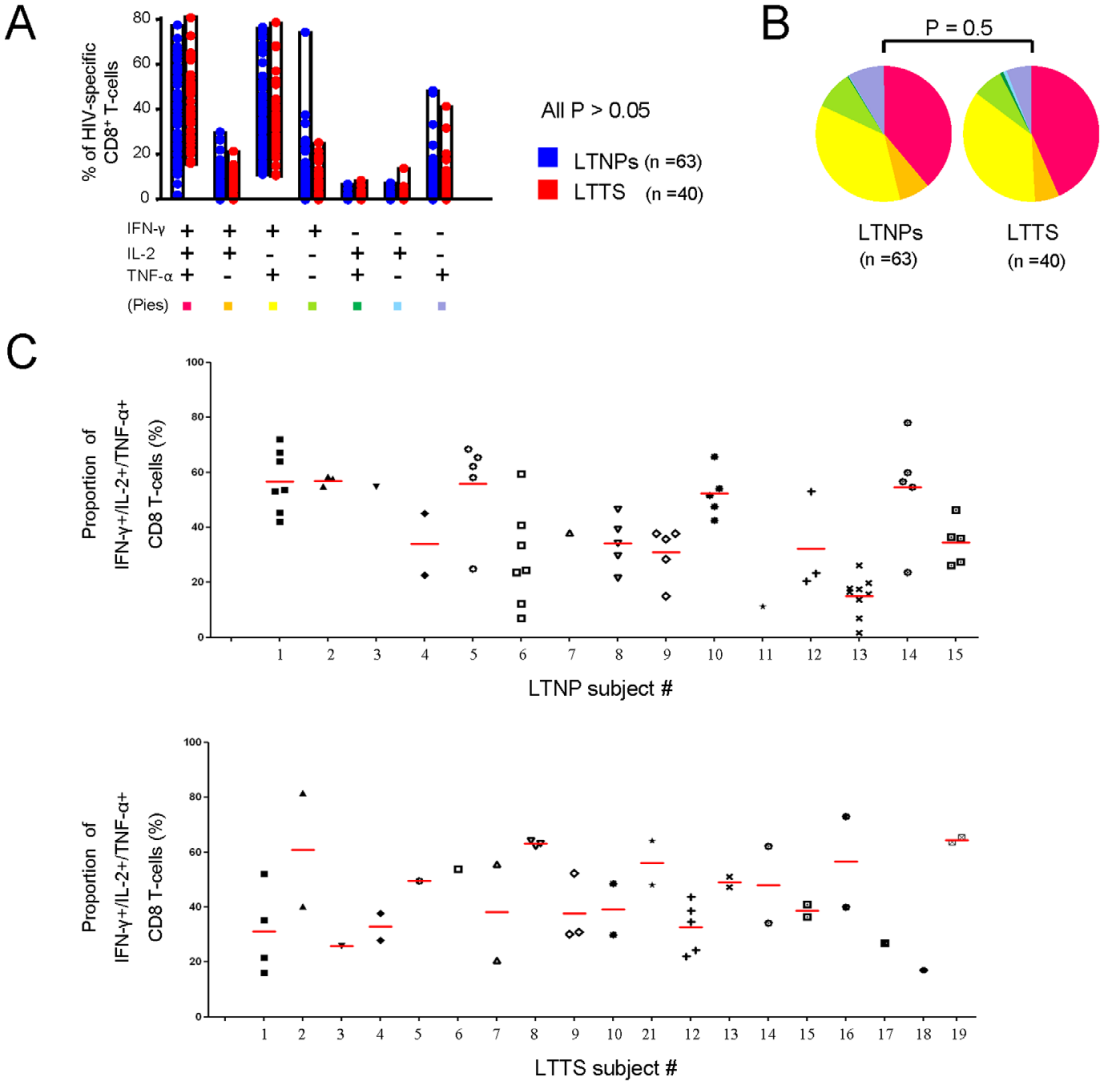
Functional cytokine profile of HIV-1-specific CD8^+^ T-cells. (**A**) Cumulative data of the simultaneous analysis of IFN-γ, IL-2 and TNF-α production. All possible combinations of IFN-γ, IL-2 and TNF-α production are shown on the x axis, whereas the percentage of the distinct cytokine-producing cell subsets within HIV-specific CD8^+^ T-cells is shown on the y axis. Pie charts (**B**) summarize the data and each slice corresponds to the proportion of HIV-1 specific CD8^+^ T-cells positive for a given combination of T-cell functions. (**C**) Per subject analysis of the proportion of CD8^+^ T-cells producing simultaneously 3 cytokines (IFN-γ + IL-2 + TNF-α). All the responses identified are shown per subject and the mean of responses is shown in red for each subject. Results from only 19 out of 20 LTTS subjects are shown as CD8^+^ T-cell responses to optimal epitopes were not identified in one subject, even though CD4^+^ and CD8^+^ T-cell responses upon stimulation with Gag peptide pool were detected. LTTS, long-term treated HIV-1 seroconverters; LTNPs: HIV-1 long-term non-progressors.

We therefore analysed and compared the HIV-1-specific CD8^+^ T-cell functional profile in LTTS and LTNPs by simultaneously analyzing IFN-γ, IL-2 and TNF-α production in the previously characterized 103 epitope-specific CD8^+^ T-cell responses. Each response was characterized for the presence of 8 possible different types of cell populations with various patterns of cytokine production. As shown in Figure 4A, both cohorts showed similar proportions of HIV-1-specific CD8^+^ T-cell populations with a given pattern of cytokine production (all P>0.05) and a similar global functional profile (P = 0.5; Figure 4B).

We next focused on the triple-positive cell population (i.e. CD8^+^ T-cells producing simultaneously IFN-γ, IL-2 and TNF-α) and restricted the analysis of polyfunctionality to this subset. As shown in Figure 4A, this population accounted on average for a similar proportion of the total HIV-1-specific CD8^+^ T-cell responses in both cohorts. Among the HIV-1-specific CD8^+^ T-cells responding to peptide stimulation, 43% and 39% were triple-positive in LTTS and LTNPs, respectively (P = 0.12). However, we observed a large variability in the level of polyfunctionality which ranged from 82% to 16% in LTTS and from 78% to 2% in LTNPs. We further analysed our data in order to verify if HIV-1-specific CD8^+^ T-cell responses with high or low proportions of triple-positive cells were detected in different subjects or within the same subjects. In other words, we assessed whether various individuals had different patterns of functional profiles or if responses with different functional profiles could be detected within the same individual. To address this issue, we analysed the level of polyfunctionality per subject (Figure 4C) and found that most subjects showed responses clustering within similar levels of triple-positive cells (i.e. LTNP subject #10 vs. #13). However, in a few patients responses differed in terms of the levels of triple-positive cells (i.e. LTNP subject #6) showing different levels of polyfunctionality of HIV-1-specific CD8^+^ T-cell responses within the same subject.

Taken together, these data demonstrate that LTTS and LTNPs display comparable levels of polyfunctional CD8^+^ T-cell responses and that factors independent of the group affiliation, i.e. LTTS or LTNPs, are associated with the level of polyfunctionality.

### Levels of HIV-1-specific CD8^+^ T-cell polyfunctionality are associated with the type of targeted epitope and HLA class I restriction

Although an overall similar degree of HIV-1-specific CD8^+^ T-cells polyfunctionality was demonstrated in the LTTS and LTNP groups, we found some variability in terms of some of the HIV-1-specific CD8^+^ T-cell responses in both groups. We therefore attempted to characterize the factors that might explain these different levels of polyfunctionality in two cohorts shown to have a similar viral burden.

We first investigated whether the observed variability in polyfunctionality was associated with the subjects’ clinical and laboratory parameters, such as age, levels of CD4^+^ and CD8^+^ T-cells, CD4^+^/CD8^+^ T-cell ratio, CD8^+^/CD38^+^ T-cell count, cell-associated HIV-1 DNA and RNA in peripheral blood, and days from diagnosis to ART initiation in LTTS. Based on simple regression analysis, none of these parameters was found to be significantly associated with the proportion of triple-positive HIV-1-specific CD8^+^ T-cells except for the level of proviral DNA in LTNPs. Of interest, levels of polyfunctionality and cell-associated HIV-1 DNA were shown to be inversely correlated in this group (P = 0.016).

**Figure 5:**
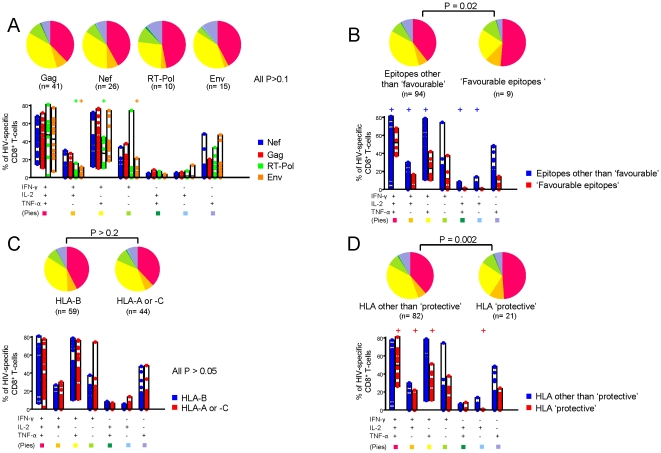
Analysis of HIV-1-specific CD8^+^ T-cell polyfunctionality. Cumulative data of the analysis of simultaneous IFN-γ, IL-2 and TNF-α production comparing CD8^+^ T-cell responses targeting distinct viral regions (**A**), targeting ‘favourable vs. ‘other than favourable’ epitopes (**B**), restricted by HLA-B vs. HLA-A or -C alleles (**C**) and restricted by HLA class I alleles associated with slow disease progression (‘protective’) vs. all others HLA class I alleles (**D**). All possible combinations of IFN-γ, IL-2 and TNF-α production are shown on the x axis, whereas the percentage of each cytokine-producing cell subset within HIV-specific CD8^+^ T-cells is shown on the y axis. Only significant differences of a given virus-specific CD8^+^ T-cell response versus all others are shown. + denotes a P value <0.05. Pie charts summarize the data, and each slice corresponds to the proportion of virus-specific CD8^+^ T-cells positive for a given combination of T-cell functions. LTTS: long-term treated HIV-1 seroconverters; LTNPs: HIV-1 long-term non-progressors.

We then considered factors described to be associated with T-cell polyfunctionality, such as the type of targeted viral region, epitope and HLA class I restriction. With regard to the type of targeted viral protein, responses against Gag have been associated with a better clinical outcome [55], even in the absence of protective HLA alleles [56]. As shown in Figure 5A, when considering various functional HIV-1-specific CD8^+^ T-cell populations individually, we found some differences among viral regions, however, the overall HIV-1-specific CD8^+^ T-cell functional profile did not differ according to the type of targeted viral protein, as demonstrated by a similar functional profile of responses targeting Gag, Nef, Env or RT-Pol proteins (all P>0.1; Figure 5A).

Recent data have also shown that the type of targeted epitope plays an important role in shaping the functional profile of HIV-1-specific CD8^+^ T-cell response and that responses targeting “favourable epitopes” are correlated with good prognosis in terms of viral control [57]. Accordingly, when pooling together all the HIV-1-specific CD8^+^ T-cell responses targeting “favourable epitopes” (n = 9) and comparing them to those that did not (n = 94), we found a different functional profile between the two types of responses and a higher degree of polyfunctionality associated with “favourable epitope”-targeting responses (P = 0.03; Figure 5B). The mean proportion of triple-positive HIV-1-specific CD8^+^ T-cells was shown to be 51.47% for “favourable epitope” (range 12.26%–68.51%) and 39.72% for non-“favourable epitope”-targeting responses (range 1.80%–81.58%) (P = 0.02; Figure 5B).

Besides the type of targeted viral protein and epitope, HLA-B restriction in general and HLA-B*5701 and B*2705 in particular have been associated with better control of HIV-1 infection [32], [34], [35], [36], [60], [61], [62]. Therefore, we analysed the level of polyfunctionality based on the type of HLA class I-restriction in our two cohorts. As shown in Figure 5C, HIV-1-specific CD8^+^ T-cell responses restricted by HLA-B alleles (n = 59) did not display a different functional profile compared to those restricted by HLA-A or C (n = 44; P>0.2). In contrast, as shown in Figure 5D, HIV-1-specific CD8^+^ T-cell responses restricted by HLA-B alleles associated with slow HIV-1 disease progression (n = 21) [37] were significantly more polyfunctional than those which were not (n = 82; P = 0.002) with a proportion of triple-positive CD8^+^ T-cells of 49% (range 37.86%–68.51%) versus 39%, respectively (range 1.79%–81.58%; P = 0.01). We found that HLA-B*5701-restricted responses (n = 15), in particular, were responsible for this higher degree of polyfunctionality. In fact, when HLA-B*5701-restricted responses were removed from the analysis, the difference in functional profile was lost (P = 0.4). It is thus possible to hypothesize that the higher level of polyfunctionality shown for responses targeting “favourable epitopes” was driven by the HLA-B*5701 restriction since 55% of these responses (5 out of 9) were HLA-B*5701-restricted.

Overall, our data demonstrate that the targeting of various viral regions was associated with a similar HIV-1-specific CD8^+^ T-cell functional profile, whereas “favourable epitope”-targeting and HLA-B*5701 restriction were associated with a higher degree of polyfunctionality.

### Ex-vivo perforin expression is low and similar in both cohorts

**Figure 6:**
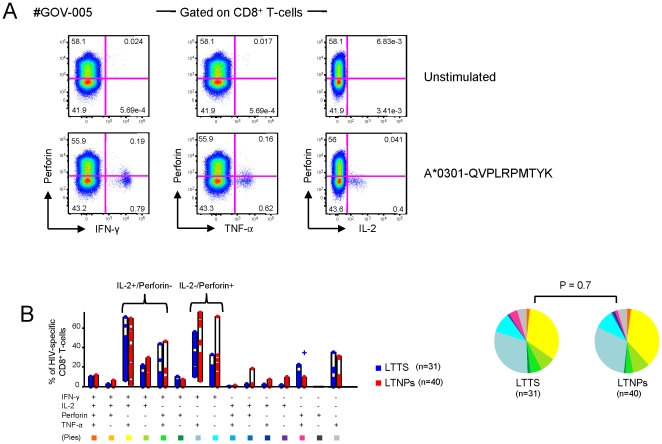
HIV-1-specific CD8^+^ T-cell perforin expression in LTTS and LTNPs. (**A**) Representative flow cytometric plots of perforin vs. IFN-γ, TNF-α and IL-2 expression are shown from one representative LTTS subject upon 6 hours *in vitro* stimulation with a Nef epitope, aa 73–82. (**B**) Cumulative data of the simultaneous analysis of perforin, IFN-γ, IL-2 and TNF-α expression. All possible combinations of perforin, IFN-γ, IL-2 and TNF-α expression are shown on the x axis, whereas the percentage of the various marker-expressing cell subsets within HIV-1 specific CD8^+^ T-cells is shown on the y axis. Only significant differences of a given virus-specific CD8^+^ T-cell response versus all the others are shown. + denotes a P value<0.05. Pie charts summarize the data, and each slice corresponds to the proportion of virus-specific CD8^+^ T-cells positive for a certain combination of T-cell functions. LTTS: long-term treated HIV-1 seroconverters; LTNPs: HIV-1 long-term non-progressors.

Cytotoxicity is the main mechanism by which CD8^+^ T-cells exert their antiviral activity with perforin as one of the most important mediators of T-cell cytotoxicity [15]. Dysfunctional perforin expression has been described at HIV seroconversion [63], however, the impact of early and prolonged treatment on this marker remains unknown. In order to compare perforin levels between LTTS and LTNPs, we have analysed its ex-vivo expression in a randomly selected subset of 24 subjects (14 LTTS and 10 LTNPs). Seventy-one optimal epitope-specific CD8^+^ T-cell responses (31 in LTTS and 40 in LTNPs) were characterized based on IFN-γ, IL-2, TNF-α and perforin expression, using the anti-perforin antibody clone B-D48 which is suitable for ICS assays. A representative example is shown in Figure 6A. The average perforin expression was found to be similarly low in both cohorts, even though a trend for a higher expression in LTNPs was observed: the mean percentage of virus-specific IFN-γ^+^ CD8^+^ T-cells expressing perforin was 0.031% (range 0.00%–0.40%) and 0.057% (range 0.00%–0.36%) in LTTS and LTNPs, respectively (P = 0.12). As shown in Figure 6B, the relative contribution of perforin to the HIV-1-specific CD8^+^ T-cell response was similar in both cohorts: the mean proportion of HIV-1-specific CD8^+^ T-cells expressing perforin was 8% in LTTS and LTNPs (range 0.00%–43.74% and 0.00%–48.26%, respectively; P = 0.45).

In addition, we have found that the dominant virus-specific CD8^+^ T-cell populations were either perforin^−^ and IL-2^−^, or perforin^+^ and IL-2^−^, or IL-2^+^ and perforin^−^ (Figure 6B). This observation confirms in HIV-1-specific CD8^+^ T-cells our previous observation in CMV-specific CD8^+^ T-cell responses of the divergent expression of perforin and IL-2, which is consistent with their respective role in cytotoxic and proliferative capacities [64].

Overall, we have demonstrated that under conditions of long-term controlled viremia, either spontaneously or with antiretroviral treatment, perforin expression on HIV-1-specific CD8^+^ T cells was low. This finding is consistent with the low antigenic exposure in both cohorts.

## Discussion

We have comprehensively characterized T-cell responses in peripheral blood in two cohorts with long-term controlled viremia in the presence or absence of ART. As such we have addressed the question of the impact of a prolonged period of successful treatment initiated very early after HIV-1 seroconversion on HIV-1-specific T-cell responses in a homogenous, laboratory-defined, single clinic-based LTTS cohort with LTNPs as a comparator. Instead of the conventionally used five parameters (i.e. IFN-γ, TNF-α, IL-2, MIP-1β and CD107a), we have focused on those which we believe are most relevant to assess HIV-1 T-cell functionality (i.e. IFN-γ, TNF-α and IL-2) and have also included the assessment of perforin expression in our analysis. Proliferative capacity and cytotoxicity have been associated with IL-2 secretion and perforin expression, respectively [9], [15], [32], [51], [52], [64], [65], [66], [67], [68]. Thus our data provide indirect information on these two key functions of antiviral CD8^+^ T-cells. We have further characterized these two cohorts in terms of levels of immune activation and viral burden.

The potential for the immune system to regenerate and avoid the damage inflicted by chronic exposure to the virus and its immunopathology with treatment initiation theoretically profoundly differentiates the acute and chronic stages of HIV-1 infection, although early immune defects have been described [63]. Short treatment periods at the time of seroconversion have been associated with some degree of HIV-1-specific T-cell preservation and reconstitution in terms of IFN-γ-producing HIV-1-specific T-cells [69]. The duration of ART-induced aviremia and nadir CD4^+^ T-cell levels have recently been shown to be important factors for CD4^+^ T-cell recovery, strongly suggesting that the timing of ART initiation and its duration are of crucial importance in terms of immune reconstitution [70]. This is supported by evidence of immunovirological control upon ART interruption in 5 HIV-infected individuals who had received very early and prolonged treatment [71].

In contrast to some recent data describing that the recovery of polyfunctionality, proliferation and cytotoxicity does not occur to the same extent in aviremic subjects on ART initiated during the chronic phase of the infection as in LTNPs [19], our results demonstrate the presence of strong polyfunctional HIV-1-specific CD4^+^ and CD8^+^ T-cell responses associated with low perforin expression in HIV-1-specific CD8^+^ T-cells in LTTS. These data are consistent with a polyfunctional/non-cytotoxic profile in a context of low viral burden as demonstrated by several virological markers used in this study. Similar results were found in LTNPs, although we observed a higher breadth and a trend towards a higher magnitude of HIV-1-specific CD8^+^ T-cell responses which were mainly driven by strong anti-Gag responses. Extensive demonstration has been made of the absence of correlation between the magnitude and breadth of HIV-1-specific IFN-γ-producing T-cells and virological control in previous studies in acute and chronic infection [72], [73], [74]. In contrast, several lines of evidence point towards the fact that slow or non-progressive HIV-1 infection, such as in LTNPs, is associated with polyfunctional HIV-1-specific T-cell responses [6], [9], [10], [74] as these have been associated with virological control in several studies. Our results therefore argue for a very substantial degree of immune preservation/reconstitution of polyfunctional HIV-1-specific T-cells with early and prolonged treatment initiation in association with low viral burden.

However, causality cannot be inferred at this stage without further evidence to prove that the type of polyfunctional HIV-1-specific T-cell responses as described in our LTNP and LTT cohorts is indeed one of the mechanisms by which virological control is achieved. Some previous data have suggested that T-cell functionality reflects antigen exposure rather than the mechanism by which viremia is controlled due to the absence of polyfunctional T-cells in some virological controllers, the possibility to induce polyfunctional responses by ART and the virologic rebound upon successful treatment cessation [21], [51], [52], [66], [75], [76], [77], [78]. However, beside its association with virological control in HIV-1 infection, a T-cell polyfunctional profile is indeed present in HIV-2 infected subjects who have an overall better prognosis than those with HIV-1 [79]. Other types of infection such as coccidiomycosis and the mouse model of *Leishmania major* infection have also shown association of control with polyfunctional T-cells [80], [81].

We are well aware that formal demonstration of the potential impact of these responses on virological rebound in LTTS would require treatment discontinuation. Previous trials with shorter duration of ART initiated at the time of PHI have not shown overall virological control except in few subjects. These subjects might have controlled plasma viremia even in the absence of treatment as it is not possible to exclude a LTNP/elite controller-type of status at this stage of the infection [73]. If indeed viral reservoirs do continue to decrease overtime with early ART initiation maintained over several years, it is tempting to imagine a situation somewhat similar to minimal residual disease in leukaemia whereby the immune system might be able to contain the infection for prolonged periods of time in some patients as recently described by Hocqueloux et al [71].

The results of the present study provide several important insights into the features of HIV-specific CD8^+^ T-cell responses in subjects with low viral burden and long-term viral control in the presence or absence of treatment. We have shown that HIV-1-specific CD8^+^ T-cell polyfunctionality was associated with the type of targeted epitope and HLA restriction, both during natural (LTNPs) and ART-induced (LTTS) viral control. Epitopes shown to be associated with slow disease progression and HLA-B*5701-restricted responses were associated with a higher degree of polyfunctionality. In contrast, the level of polyfunctionality did not differ among HIV-1-specific CD8^+^ T-cell responses targeting other viral proteins.

We have described in a previous study [15] that perforin expression is modulated *in vitro* and *in vivo* by antigenic exposure. Consistently, we have confirmed here that under conditions of similar low viral burden the ex-vivo expression of perforin is low. The trend for higher perforin expression in LTNPs as compared to LTTS is in line with data from a recent study [22]. One could speculate that the lower level of perforin observed in our LTTS cohort was due to ART-mediated control of viremia in contrast to LTNPs where further immune-based mechanisms may be needed to control viremia in the absence of treatment. However, our data do not allow us to formally exclude a residual decrease in perforin expression in treated seroconverters, even after a prolonged ART period.

The overall slightly lower levels of perforin expression by HIV-1-specific CD8^+^ T-cells observed in our study compared to those found by Hersperger and colleagues [22] are possibly due to differences in viral burden between the two cohorts as aviremia might have been more prolonged in our LTNP subjects (11 subjects with permanent aviremia at <50 and 4 subjects with <1000 HIV-1 RNA copies/mL in at least 90% measurements over 7 years vs. <75 or <50 HIV-1 RNA copies/mL in at least 3 measurements over 1 year, respectively). Consistent with the lower perforin expression, IL-2 production was about four times higher in our LTNP cohort compared with the other study. We [64] and others [82] have indeed shown that IL-2 and perforin expression by virus-specific CD8^+^ T-cells is divergent.

We have added for comparative purposes between our two cohorts further virological markers beside plasma viremia measurements such as cell-associated HIV-1 DNA and RNA which are aimed at better characterizing viral burden in peripheral blood. We could show that both cohorts were not different in terms of cell-associated HIV-1 DNA and RNA levels in blood, in contrast to what has recently been described in subjects who had been treated in chronic infection for a similar duration as our LTTS cohort [54]. However, caution should be applied in the interpretation of viral reservoir levels in terms of viral rebound as it might still occur upon treatment cessation even in patients with extremely low viral burden [83]. The low level of reservoirs in LTTS suggests that long-term ART is associated with a decrease of their saturation overtime as previously described [42], [84] although we do not have longitudinal virological data from our cohort to conclusively prove it and we have not investigated the extent of gut reservoirs. Our results argue in favour of either an incomplete saturation of reservoirs at the time of initiation of ART at PHI or a more rapid decrease as compared to ART-treated chronic infection as suggested by Chun et al [41].

In summary, our results demonstrate that subjects treated with ART for several years at the time of seroconversion show great similarity with LTNPs in terms of polyfunctional HIV-1-specific T-cell responses, level of immune activation, residual viral replication and saturation of HIV-1 reservoirs in peripheral blood. These data should help define new types of interventions aimed at optimizing ART timing and duration and implementing novel therapeutic interventions in order to further deplete peripheral reservoirs, enhance protective cellular immune responses and promote virological control post-stopping treatment.

## Supporting Information

Data S1 **Gating strategy for identification of CD4^+^ and CD8^+^ T-cells.** Shown is a representative example of HIV-1 Gag-specific responses from LTTS subject JIM-014 upon 6-hour *in vitro* stimulation with the Gag peptide pool. This figure illustrates the gating strategy used in the comprehensive analysis of cytokine production and cytotoxic capacity as measured by IFN-γ, IL-2 and TNF-α production and perforin expression, respectively, in the ICS assay. After initial gating on lymphocytes using forward and side scatter properties, gating on forward scatter area (FSC-A) versus height (FSC-H) was used to remove doublets. Events were further gated on IFN-γ versus the dead cell marker to remove dead cells. CD3^+^ T-cells were gated on the remaining live cells. CD3^+^CD8^+^ T-cells were selected based on CD8^+^ staining and CD4^+^ T-cells were then excluded. Cells within these individual response gates were then entered into Boolean gating analysis to generate frequencies for all possible combinations (i.e., positive or negative) of the distinct functions (FlowJo software, version 8.8.2; TreeStar). LTTS: long-term treated HIV-1 seroconverters; LTNPs: HIV-1 long-term non-progressors.
(PPT)

Data S2 **Analysis of the magnitude of HIV-1-specific CD8^+^ T-cell responses. (A)** Cumulative data (mean±SE) on the percentage of IFN-γ-, IL-2- and TNF-α-producing HIV-1 specific CD8^+^ T-cells following 6 hours of *in vitro* stimulation with ‘favourable’ epitopes (i.e. optimal CD8^+^ T-cell epitopes known to be associated with good viral control). (**B**) Cumulative data (mean±SE) on the percentage of IFN-γ, IL-2 and TNF-α production in HLA-B*5701-restricted CD8^+^ T-cell responses. LTTS: long-term treated HIV-1 seroconverters; LTNPs: HIV-1 long-term non-progressors.
(PPT)
