# Supplementary material for: Early and Prolonged Antiretroviral Therapy Is Associated with an HIV-1-Specific T-Cell Profile Comparable to That of Long-Term Non-Progressors
Source: PLoS One. 2011 Apr 5;6(4):e18164. doi: 10.1371/journal.pone.0018164 (PMC3071718; doi:10.1371/journal.pone.0018164)
Supplement: Data S1 — Gating strategy for identification of CD4+ and CD8+ T-cells. Shown is a representative example of HIV-1 Gag-specific responses from LTTS subject JIM-014 upon 6-hour in vitro stimulation with the Gag peptide pool. This figure illustrates the gating strategy used in the comprehensive analysis of cytokine production and cytotoxic capacity as measured by IFN-γ, IL-2 and TNF-α production and perforin expression, respectively, in the ICS assay. After initial gating on lymphocytes using forward and side scatter properties, gating on forward scatter area (FSC-A) versus height (FSC-H) was used to remove doublets. Events were further gated on IFN-γ versus the dead cell marker to remove dead cells. CD3+ T-cells were gated on the remaining live cells. CD3+CD8+ T-cells were selected based on CD8+ staining and CD4+ T-cells were then excluded. Cells within these individual response gates were then entered into Boolean gating analysis to generate frequencies for all possible combinations (i.e., positive or negative) of the distinct functions (FlowJo software, version 8.8.2; TreeStar). LTTS: long-term treated HIV-1 seroconverters; LTNPs: HIV-1 long-term non-progressors. (PPT) [file pone.0018164.s001.ppt]

## Slide 1
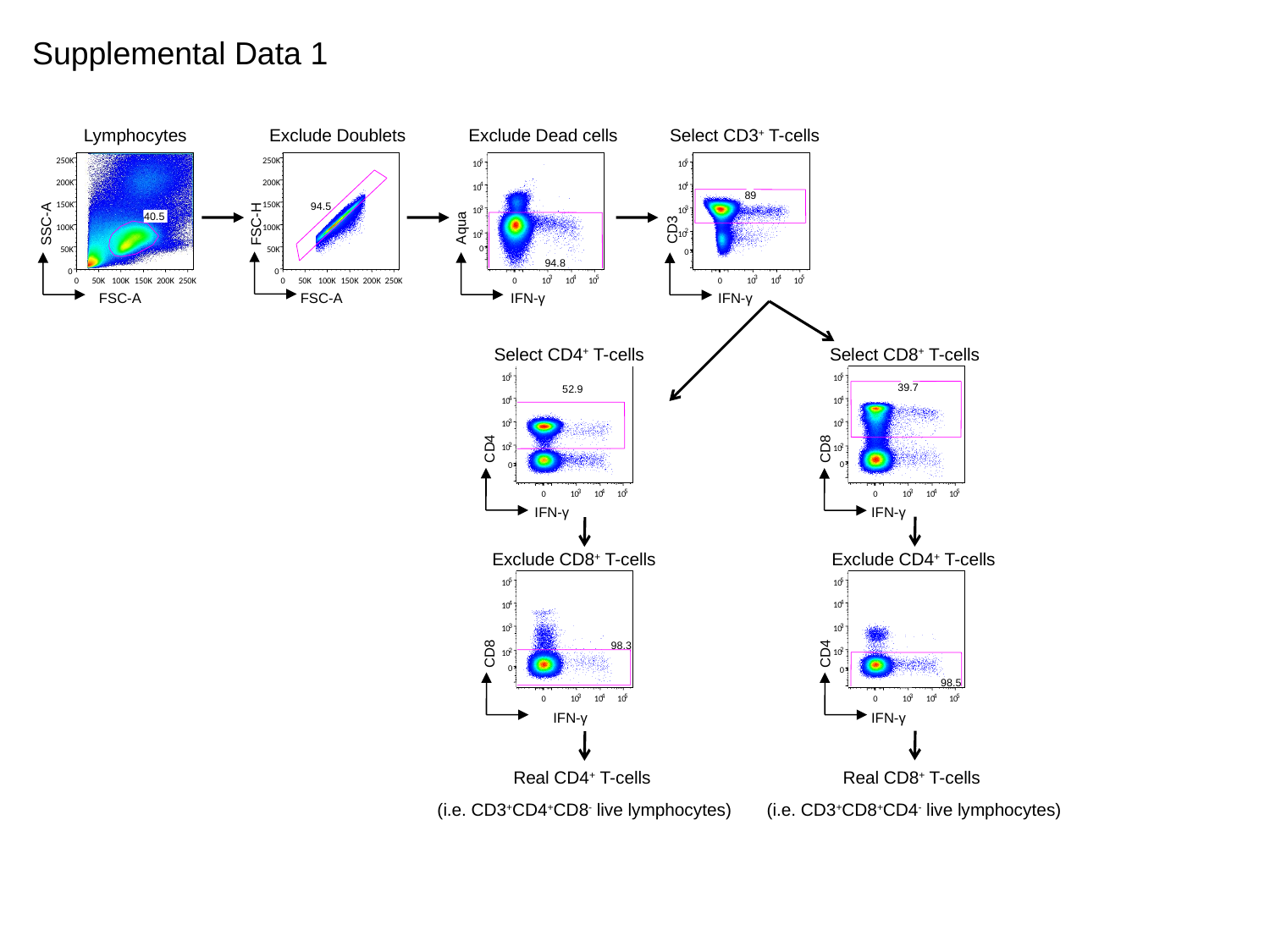

Supplemental Data 1
Lymphocytes
Exclude Doublets
Exclude Dead cells
Select CD3+ T-cells
40.5
250K
200K
150K
100K
50K
0
0
50K
100K
150K
200K
250K
94.5
250K
200K
150K
100K
50K
0
0
50K
100K
150K
200K
250K
5
10
4
10
3
10
2
10
0
94.8
3
4
5
0
10
10
10
89
5
10
4
10
3
10
2
10
0
3
4
5
0
10
10
10
SSC-A
FSC-H
Aqua
CD3
FSC-A
FSC-A
IFN-γ
IFN-γ
Select CD4+ T-cells
Select CD8+ T-cells
39.7
5
10
4
10
3
10
2
10
0
3
4
5
0
10
10
10
52.9
5
10
4
10
3
10
2
10
0
3
4
5
0
10
10
10
CD4
CD8
IFN-γ
IFN-γ
Exclude CD8+ T-cells
Exclude CD4+ T-cells
5
10
4
10
3
10
2
10
0
98.3
3
4
5
0
10
10
10
5
10
4
10
3
10
2
10
0
98.5
3
4
5
0
10
10
10
CD8
CD4
IFN-γ
IFN-γ
Real CD4+ T-cells
(i.e. CD3+CD4+CD8- live lymphocytes)
Real CD8+ T-cells
(i.e. CD3+CD8+CD4- live lymphocytes)
