# Supplementary material for: Early and Prolonged Antiretroviral Therapy Is Associated with an HIV-1-Specific T-Cell Profile Comparable to That of Long-Term Non-Progressors
Source: PLoS One. 2011 Apr 5;6(4):e18164. doi: 10.1371/journal.pone.0018164 (PMC3071718; doi:10.1371/journal.pone.0018164)
Supplement: Data S2 — Analysis of the magnitude of HIV-1-specific CD8+ T-cell responses. (A) Cumulative data (mean±SE) on the percentage of IFN-γ-, IL-2- and TNF-α-producing HIV-1 specific CD8+ T-cells following 6 hours of in vitro stimulation with ‘favourable’ epitopes (i.e. optimal CD8+ T-cell epitopes known to be associated with good viral control). (B) Cumulative data (mean±SE) on the percentage of IFN-γ, IL-2 and TNF-α production in HLA-B*5701-restricted CD8+ T-cell responses. LTTS: long-term treated HIV-1 seroconverters; LTNPs: HIV-1 long-term non-progressors. (PPT) [file pone.0018164.s002.ppt]

## Slide 1
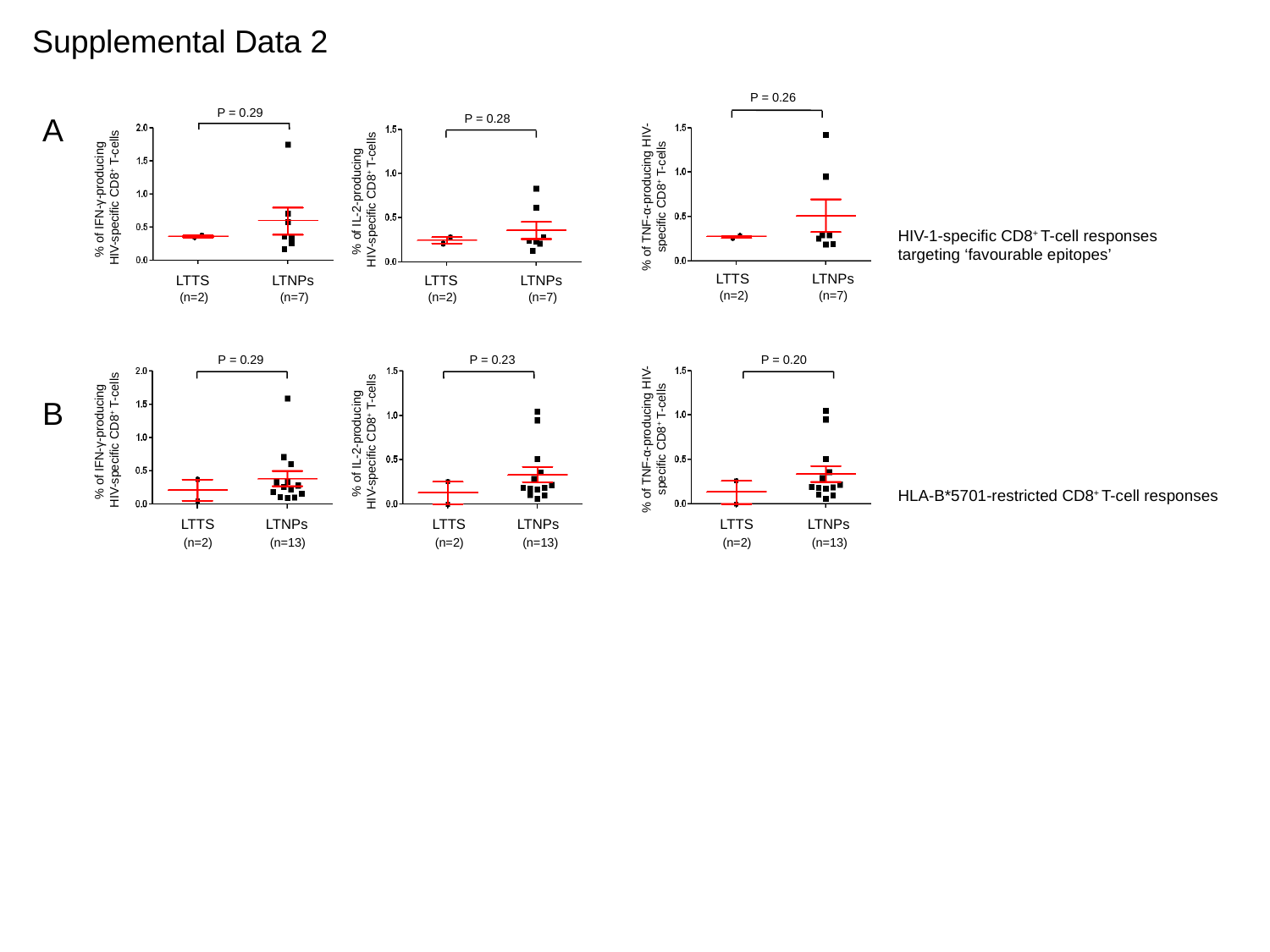

Supplemental Data 2
P = 0.26
P = 0.29
A
P = 0.28
% of TNF-α-producing HIV-specific CD8+ T-cells
% of IFN-γ-producing
HIV-specific CD8+ T-cells
% of IL-2-producing
HIV-specific CD8+ T-cells
HIV-1-specific CD8+ T-cell responses targeting ‘favourable epitopes’
LTTS
LTNPs
LTTS
LTNPs
LTTS
LTNPs
(n=2)
(n=7)
(n=2)
(n=7)
(n=2)
(n=7)
P = 0.29
P = 0.23
P = 0.20
B
% of TNF-α-producing HIV-specific CD8+ T-cells
% of IFN-γ-producing
HIV-specific CD8+ T-cells
% of IL-2-producing
HIV-specific CD8+ T-cells
HLA-B*5701-restricted CD8+ T-cell responses
LTTS
LTNPs
LTTS
LTNPs
LTTS
LTNPs
(n=2)
(n=13)
(n=2)
(n=13)
(n=2)
(n=13)
